# XPC Protein Improves Lung Adenocarcinoma Prognosis by Inhibiting Lung Cancer Cell Stemness

**DOI:** 10.3389/fphar.2021.707940

**Published:** 2021-11-03

**Authors:** Weiyu Wang, Shengyao Ma, Zhenyu Ding, Yang Yang, Huaijie Wang, Kunning Yang, Xiaoshan Cai, Hanyue Li, Zhiqin Gao, Meihua Qu

**Affiliations:** ^1^ Translational Medical Center, Weifang Second People’s Hospital, The Second Affiliated Hospital of Weifang Medical University, Weifang, China; ^2^ Biopharmaceutical Laboratory, Key Laboratory of Shandong Province Colleges and Universities, School of Life Science and Technology, Weifang Medical University, Weifang, China; ^3^ College of Pharmacy, Weifang Medical University, Weifang, China; ^4^ Department of Orthopedics, Shanghai Jiao Tong University Affiliated Sixth People’s Hospital, Shanghai, China; ^5^ School of Public Health, Qingdao University, Qingdao, China

**Keywords:** XPC, lung cancer stem cell biomarkers, lung adenocarcinoma, prognostic, metastasis

## Abstract

**Objective:** Xeroderma Pigmentosum Complementation Group C (XPC) is a protein involving in nucleotide excision repair (NER). XPC also plays an important role in the lung cancer occurrence with the mechanism remian unclear up to date. Studies showed that the increased stemness of lung cancer cells is related to the recurrence and metastasis of lung cancer. This study aimed to study and analyze the correlation of XPC with lung cancer stem cell biomarkers expression and the overall survival (OS) of lung adenocarcinoma patients.

**Methods:** 140 cases of clinical lung adenocarcinoma tissue samples and 48 cases of paired paracancerous tissue samples were made into tissue microarray. Immunohistochemistry (IHC) was used to detect the expression of XPC and CD133 in cancer and paracancerous tissues. Semi-quantitative analysis and statistics were performed by Pannoramic Digital Slide Scanner. The expression of XPC and CD133 in fresh tissues was verified by Western blotting assay. siXPC was used to knock down XPC in lung cancer cell lines to study the effect of XPC on the expression of lung cancer stem cell biomarkers and the ability of cell invasion. And shXPC was used to knockdown XPC in A549 and H1650 to study the effect of XPC on the expression of lung cancer stem cell biomarkers.

**Results:** IHC and Western blotting results showed that XPC expression significantly decreased, while CD133 expression significantly increased in cancer tissues comparing to paracancerous tissues (*P*_*XPC*_ < 0.0001, *P*_*CD133*_ = 0.0395). The high level of XPC in cancer was associated with a better prognosis (*Log-rank p* = 0.0577) in lung adenocarcinoma patients. Downregulation of XPC in lung cancer cells showed increased expression of cancer stem cell biomarkers and the increased cell invasion abilities.

**Conclusion:** It is suggested that XPC can exert the ability of anti-tumor formation, tumor invasion and metastasis inhibition, and prognostic survival improvement in lung adenocarcinoma patients by regulating the stemness of lung cancer cells.

## Introduction

Lung cancer is the malignant tumor with the highest mortality and the main cause of cancer-related death worldwide, which can be divided into non-small cell lung cancer (NSCLC) and small cell lung cancer (SCLC) according to different histological subtypes (Siegel et al., 2020). NSCLC is the main type of lung cancer, accounting for about 85% (Heng et al., 2018). Histologically, NSCLC can be divided into lung adenocarcinoma, lung squamous cell carcinoma, and large cell lung cancer (Cui et al., 2015; Bray et al., 2018; Ferlay et al., 2018). Lung adenocarcinoma accounts for more than 40% of NSCLC and is the most common histological subtype of lung cancers (Xu et al., 2020). Although new treatment strategies, including molecularly targeted drugs and immune checkpoint inhibitors, have been developed, the average 5-years survival of lung cancer patients is only 16.6% due to the recurrence and metastasis of lung cancer and the individual’s resistance to cytotoxic drugs (Howlader et al., 2012; Gholami et al., 2019).

XPC is a DNA damage repair protein, which plays an important role in the process of nucleotide excision repair (NER) (Thoma and Vasquez, 2010) which repairs DNA damage caused by various physical and chemical effects including ultraviolet radiation, cigarette exposure, and chemotherapeutic drugs (Reed, 1998). Studies showed that low expression or mutation of XPC positively is related to cancer occurrence and drug resistance during cancer treatment (Teng et al., 2019). Analysis of TCGA data showed that the expression of XPC was low in human lung adenocarcinoma cancer tissues compared with paracancerous tissues (http://ualcan.path.uab.edu/cgi-bin/TCGAExResultNew2.pl?genenam=XPC&ctype=LUAD). The overexpression of XPC reduced the cytotoxic effect of platinum-based chemotherapeutic drugs such as cisplatin and rendered lung cancer cells resistant to the platinum-based chemotherapeutic drugs (Teng et al., 2019; Liu et al., 2020).

Cancer stem cells (CSCs) are believed to be involved in tumor invasion and metastasis, including lung adenocarcinoma (Charafe-Jauffret et al., 2009; Jiao et al., 2010; Xin et al., 2013; Chen et al., 2014). Clinical data indicated that overexpression of CSCs molecular biomarkers is related to poor prognosis of patients (Eppert et al., 2011). Epithelial-Mesenchymal Transition (EMT) induced by knocking out E-Cadherin showed higher expression of stem cell biomarker OCT4, increased cell migration and invasion in cervical cancer cells (Sharma et al., 2021). Wang et al.’s studies showed a decreased expression of E-cadherin when knocking down or knocking out XPC in lung cancer cells (Cui et al., 2015). We hypothesized that downregulation of XPC could increase the expression of lung cancer stem cell biomarkers and the cancer cell stemness, thereby affecting the occurrence of lung cancer and prognosis of lung cancer patients.

## Materials and Methods

### Specimens

A total of 140 tissue samples from patients with lung adenocarcinoma who underwent surgical treatment at Weifang Second People’s Hospital of City from January 1, 2011, to December 31, 2015, were collected. Tissue microarray chips containing these 140 cases of lung adenocarcinoma tissues and 48 cases of corresponding paracancerous tissue were prepared by Shanghai Outdo Biotech Co., Ltd (Shanghai, China). The clinical and pathological information of the tissue samples was obtained from the hospital information system. The OS of the patients was followed up by telephone in August 2020.

Eight fresh lung cancer specimens and paracancerous specimens were collected in Weifang Second People’s Hospital from July 1 to 31, 2020. After the surgery, the fresh tissues separated by the pathologist from cancer tissues and paracancerous tissues were washed with saline to remove blood, and stored in a −80°C refrigerator.

This study was approved by the Ethics Committee of Weifang Medical University and Weifang Second People’s Hospital. All participants were competent to provide their informed consent.

### Cell Culture

Human lung cancer A549, H1650, and H1299 cells were obtained from the National Biomedical Laboratory Cell Resource Bank (Beijing, China). A549 cells were maintained in McCoy’s 5A Media (Gibco) with 10% fetal bovine serum (FBS) (Sijiqing, Zhejiang Tianhang Biological Technology Co., Ltd.), 100 μg/ml streptomycin, and 100 units/ml penicillin. NCI-H1650 and NCI-H1299 cells were maintained in RPMI 1640 basic medium with 10% FBS, 100 μg/ml streptomycin, and 100 units/ml penicillin.

### Tissue Microarray and Immunohistochemistry

IHC was performed in the tissue microarray slides. The tissue microarray slice was cut into 4um, baked in an oven at 60°C for 60 min. After dewaxing, hydrogen peroxide blocking, citric acid (BL604A, Biosharp) antigen retrieval, and goat serum (SL038, Solarbio) blocking for 30 min at 37°C, the tissue slides were incubated with the primary rabbit antibody against XPC (1:500) (ab155025, Abcam) and rabbit antibody against CD133 (1:1000) (ab19898, Abcam) overnight at 4°C. Slides were then incubated with HRP-conjugated secondary antibody at room temperature for 2 h. Fresh 3, 3-diaminobenzidine (DAB) solution was used to visualize the target proteins, and hematoxylin was used as a tissue counterstain. The images were scanned with a Pannoramic Digital Slide Scanner (3DHISTECH) and analyzed using Pannoramic Viewer software (3DHISTECH).

The expression of XPC and CD133 in IHC images was scored independently by two pathologists. The total score is the sum of the positive cell count score and the positive color intensity score. The number of positive cells score was assigned as follows: 3, staining in ≥50% of tumor cells; 2, staining in ≥25% of tumor cells; 1, staining in ≥1% of tumor cells; and 0, no staining. The positive color intensity score was assigned as follows: 3, strong positive; 2, medium positive; 1, weak positive; and 0, no staining. The tumor tissues with a total score ≥5 points were high expression (XPC+, CD133+), and those with a total score <5 points were low expression (XPC−, CD133−).

### Western Blotting Analysis

Proteins extracted from fresh tissues (lung adenocarcinoma cancer tissue and paracancerous tissues) and cells were subjected to 10% sodium dodecyl sulfate-polyacrylamide gel electrophoresis (SDS-PAGE) and then transferred onto Immobilon-P Membrane (IPVH100010–26.5*3.75, Millipore). The membranes were blocked with tris-buffered saline with Tween 20 containing 5% non-fat milk for 2.5 h at room temperature. After washing by TBST, and followed by incubation with rabbit polyclonal anti-CD133 (1:1000) (ab19898, Abcam), mouse monoclonal anti-OCT4 antibody (1:1000) (ab184665, Abcam), rabbit polyclonal anti-XPC antibody (1:1000) (ab155025, Abcam), rabbit monoclonal anti-GAPDH antibody (1:1000) (#5174, Cell Signaling Technology) rabbit monoclonal anti-β-Actin antibody (1:1000) (#4970,Cell Signaling Technology) overnight at 4°C. The following day, the membranes were incubated with secondary goat anti-rabbit H&L (HRP) (ab6721, Abcam) at room temperature for 2 h. Protein bands were developed using the ECL Western blotting Substrate (PE0010, Solarbio) for about 10 s, and then visualized on the Western blotting detection system (Bio-Rad, United States).

### siRNA/shRNA and Transfection

siXPC/shXPC and a scramble non-targeting siRNA/shRNA (siCtrl/shCtrl) were purchased from Gene Pharma (Shanghai, China). The sequence of siCtrl is 5′-UUC​UCC​GAA​CGU​GUC​ACG​U-3′, siXPC is 5′-GCAAAUGGC UUCUAUCGAA-3′, shCtrl is 5′-TTC​TCC​GAA​CGT​GTC​ACG​T-3′, and the sequence of shXPC is 5′-CCG​GCC​CAC​TGC​CAT​TGG​CTT​ATA​TCT​CGA​GAT​ATA​A GCC​AAT​GGC​AGT​GGG​TTT​TTG-3′. The siXPC and siCtrl were transfected into cells using Lipofectamine 2000 reagents from Thermo Fisher (MA, United States) according to the manufacturer’s instructions. The shXPC and shCtrl were transfected into cells using polybrene at a final concentration of 5 μg/ml from Gene Pharma (Shanghai, China).

### Flow Cytometry

Cells were separated into shCtrl and shXPC groups, and incubated with anti-human CD133 (CD133/1) PE (130–113-670, Meltenyi Biotec, Germany) and anti-human OCT4 (OCT3) FITC (653705, Biolegend, United States) for 30 min at 4°C. The staining cells were analyzed using a flow cytometer (Beckman Coulter, United States).

### Cell Migration Assay

The cells were seeded into 6-well plates with 2.5×10^5^ cells/well. On the second day, cells were transfected and a cell scratch experiment was performed with a 200 ul pipette tip. Images at 0 and 48 h after the scratch were taken. The cell migratory ability was evaluated.

### Xenograft Tumor Growth

NU/NU Nude Mouse (6–8 weeks, male, 20–25 g body weight) was obtained from Beijing Vital River Laboratory Animal Technology Co., Ltd. (Beijing, China). Animals were maintained following institutional policies, and all studies were performed with the approval of the IACUC at Weifang Second People’s Hospitals. To generate xenografts, 3×10^6^ cells were mixed with PBS and injected subcutaneously into the upper right back of each mouse. Tumor growth was measured using calipers, and volumes were calculated based on the or mula V = (a × b^2^)/2, in which a is the longest and b is the shortest diameter of the tumor. At the end of the experiment, the animals were euthanized, and the tumor mass was harvested and photographed.

### Statistical Analysis

The expression statistics of XPC and CD133 in different pathological characteristics of lung adenocarcinoma were compared between groups with IBM SPSS Statistics 22. The expression difference of XPC and CD133 in cancer and paracarcancerous tissues was statistically analyzed by GraphPad Primes 8 software using T-test (and nonparametric tests). The relationship between XPC and CD133 and prognosis were analyzed by GraphPad Primes 8 software using a survival curve. The expression of stem cell biomarkers in two groups of 3 cell lines (siCtrl group and siXPC) was compared in pairs with IBM SPSS Statistics 22. All experiments were repeated three times, and SD represents the degree of dispersion between each experiment.

## Results

### Demographic Features of Patients

In this study, the basic information and pathological conditions of 140 samples were obtained from the Hospital Information System (HIS), including the patient’s age, gender, TNM stage, location of the primary lung cancer site, grade of differentiation, and smoking status. Among these patients are 79 males (56.43%) and 61 females (43.57%). There are more male patients with lung cancer than females, which may be attributed to smoking. The average age of all the patients was 59.58 years old (40–79 years old, 59.58 ± 8.28 years old), and most of the patients were middle-aged and elderly. The basic demography of these 140 lung adenocarcinoma patients is shown in Table 1. XPC and CD133 expression according to patient characteristics and tumor pathological features are presented in Table 2. XPC expression was significantly associated with subjects with lower age (*p* = 0.045) and lower lymph node involvement (*p* = 0.050). No correlations between XPC expression and gender, tumor size, or TNM stage. The expression of CD133 showed no significant correlation with age, gender, TNM stage, tumor size, lymph node involvement, or metastasis analyzed based on the 140 tissues of lung adenocarcinoma.

**TABLE 1:** Basic pathological information of 140 cases of lung adenocarcinoma.

Variable	Intratumor, n (%) (N = 140)	Peritumor, *n* (%) (N = 48)
Age (years)		
41–50	23 (16.4%)	9 (18.8%)
51–60	54 (38.6%)	15 (31.2%)
61–70	51 (36.4%)	21 (43.8%)
70+	12 (8.6%)	3 (6.2%)
Gender		
Male	79 (56.4%)	24 (50%)
Female	61 (43.6%)	24 (50%)
TNM Staging		
Ⅰ	57 (40.7%)	29 (60.4%)
Ⅱ	20 (14.3%)	5 (10.4%)
Ⅲ	20 (14.3%)	2 (4.2%
Ⅳ	14 (10.0%)	4 (8.3%)
Unclassified	29 (20.7%)	8 (16.7%)
Tumor Size		
T1	32 (22.9%)	14 (29.2%)
T2	61 (43.6%)	25 (52.1%)
T3	9 (6.4%)	1 (2.1%)
T4	9 (6.4%)	0 (0%)
Unknown	29 (20.7%)	8 (16.6%)
Lymph Node Involvement		
N0	83 (59.3%)	35 (72.9%)
N1	7 (5.0%)	1 (2.1%)
N2	17 (12.1%)	2 (4.2%)
N3	4 (2.9%)	2 (4.2%)
Unknown	29 (20.7%)	8 (16.6%)
Metastasis		
M0	97 (69.3%)	36 (75.0%)
M1	14 (10.0%)	4 (8.3%)
Unknown	29 (20.7%)	8 (16.7%)
Differentiation		
Well differentiated	20 (14.3%)	11 (22.9%)
Moderate differentiated	74 (52.9%)	23 (47.9%)
Poorly differentiated	29 (20.7%)	11 (22.9%)
Unknown	17 (12.1%)	3 (6.3%)
Tumor primary site		
Right upper lobe	53 (37.9%)	22 (45.8%)
Right middle lobe	10 (7.1%)	5 (10.4%)
Right lower lobe	32 (22.9%)	10 (20.8%)
Right lung whole lobe	2 (1.4%)	0 (0%)
Left upper lobe	18 (12.9%)	6 (1.25%)
Left lower lobe	20 (14.3%)	55 (10.4%)
Left lung whole lobe	5 (3.6%)	0 (0%)
Smoking		
Yes	51 (36.4%)	16 (33.3%)
No	78 (55.7%)	31 (64.6%)
Unknown	11 (7.9%)	1 (2.1%)

**TABLE 2:** Expression of XPC and CD133 in lung adenocarcinoma.

Variable	XPC expression		P	CD133 expression		P
	High	Low		High	Low	
Age (years)			0.045			0.818
41–50	5 (8.5%)	12 (15.0%)		5 (29.4%)	12 (70.6%)	
51–60	25 (42.4%)	21 (26.3%)		16 (34.8)	30 (65.2%)	
61–70	20 (33.9%)	41 (51.2%)		17 (28.8%)	42 (71.2%)	
70+	9 (15.3%)	6 (7.5%)		6 (40.0%)	9 (60.0%)	
Gender			0.155			0.558
Male	29 (37.2%)	49 (62.8%)		26 (34.2%)	50 (65.8%)	
Female	30 (49.2%)	31 (50.8%)		18 (29.5%)	43 (70.5%)	
TNM Staging			0.374			0.325
I	16 (39.0%)	25 (61.0%)		11 (27.5%)	29 (72.5%)	
II	19 (55.9%)	15 (44.1%)		10 (30.3%)	23 (69.7%)	
III	7 (35.0%)	13 (65.0%)		10 (50.0%)	10 (50.0%)	
IV	6 (40.0%)	9 (60.0%)		6 (40.0%)	9 (60.0%)	
Unclassified	11 (37.9%)	18 (62.1%)		7 (24.1%)	22 (75.9%)	
Tumor Size			0.869			0.157
T1	12 (37.5%)	20 (62.5%)		10 (31.3%)	22 (68.8%)	
T2	28 (46.7%)	32 (53.3%)		17 (29.3%)	41 (70.7%)	
T3	4 (44.4%)	5 (55.6%)		4 (44.4%)	5 (55.6%)	
T4	4 (44.4%)	5 (55.6%)		6 (66.7%)	3 (33.3%)	
Unknown	11 (37.9%)	18 (62.1%)		7 (24.1%)	22 (75.9%)	
Lymph Node Involvement			0.050			0.169
N0	36 (43.4%)	47 (56.6%)		26 (32.1%)	55 (67.9%)	
N1	5 (100.0%)	0 (0.0%)		0 (0.0%)	5 (100%)	
N2	6 (33.3%)	12 (66.7%)		9 (50.0%)	9 (50.0%)	
N3	1 (25.0%)	3 (75.0%)		2 (50.0%)	2 (50.0%)	
Unknown	11 (37.9%)	18 (62.1%)		7 (24.1%)	22 (75.9%)	
Metastasis			0.371			0.512
M0	37 (38.5%)	59 (61.5%)		31 (33.3%)	62 (66.7%)	
M1	8 (53.3%)	7 (46.7%)		6 (40.0%)	9 (60.0%)	
Unknown	14 (50.0%)	14 (50.0%)		7 (24.1%)	22 (75.9%)	

### XPC is Low Expressed in Lung Adenocarcinoma Cancer Tissue

**FIGURE 1:**
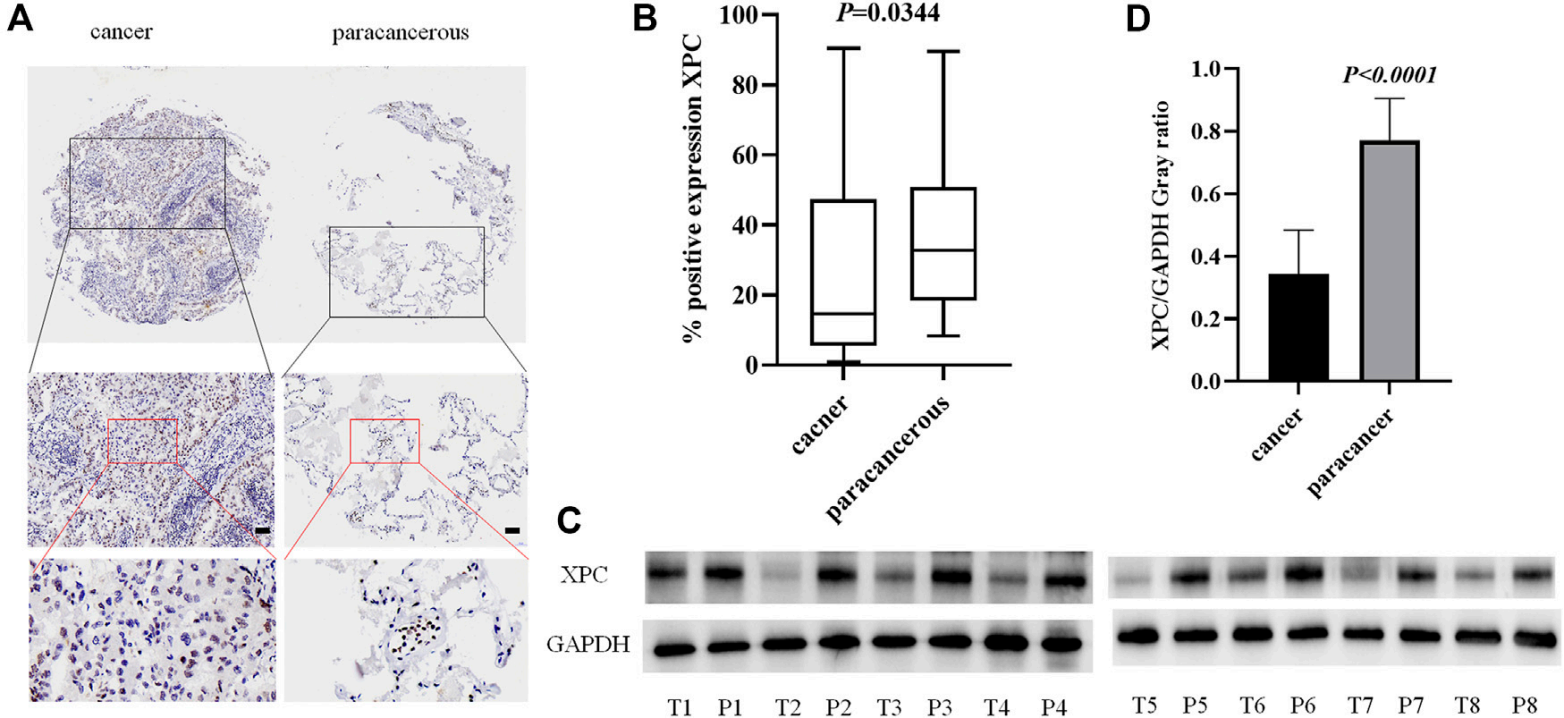
The expression of XPC in lung adenocarcinoma cancer tissue and paracancerous tissue. **(A)**. IHC detection of XPC expression in lung adenocarcinoma cancer tissue and paracancerous tissue. Brown means XPC positive reaction, XPC is expressed in the nucleus, and blue is nuclear staining. All bar in the figure = 50 μm. **(B)**. GraphPad Primes 8 statistics found that XPC expression is higher in paracancerous tissue than in cancer tissue (*p* = 0.0344). **(C)**. T is cancer tissue, P is paracancerous tissue. Western blot detects the expression of XPC in the fresh cancer tissue and paracancerous tissue of 8 lung adenocarcinoma patients. **(D)**. Western blot counts the expression of XPC in fresh cancer tissue and paracancerous tissue of 8 lung adenocarcinoma patients, and the expression in paracancerous tissue is higher than that in cancer tissue (*p* < 0.0001). Bars represent the means ± SD of 8 lung adenocarcinoma patients.

To detect the expression of XPC in lung adenocarcinoma cancer and paracancerous tissues, we used tissue microarrays with 48 pairs of lung adenocarcinoma cancer and paracancerous tissues for IHC staining. The results showed that XPC is located in the cell nucleus and is highly expressed in lung adenocarcinoma paracancerous tissue while low in cancer tissue (*p* = 0.0344) (Figures 1A,B). To further determine the protein expression level of XPC in cancer tissue and paracancerous tissue, we collected fresh lung adenocarcinoma cancer tissue and paracancerous tissue for Western blot analysis. Similar to the IHC results, the expression of XPC is lower in lung adenocarcinoma cancer tissue compared to the corresponding paracancerous tissue (*P*_*WB*_ < 0.0001) (Figures 1C,D). These results suggested that the low expression of XPC in cancer tissues may be related to the occurrence of lung adenocarcinoma.

### CD133 is Overexpressed in Lung Adenocarcinoma Cancer Tissue

**FIGURE 2:**
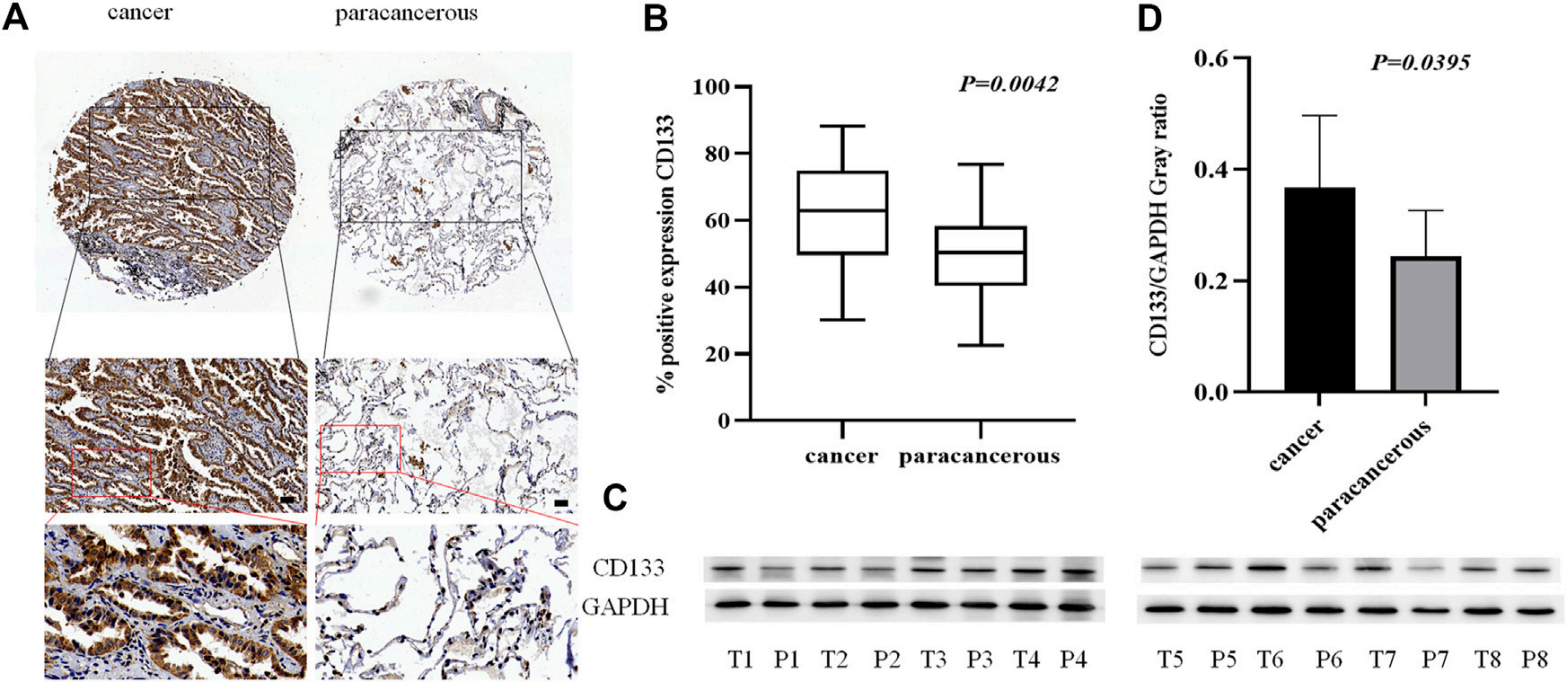
The expression of CD133 in lung adenocarcinoma cancer tissue and paracancerous tissue. **(A)**. IHC detection of CD133 expression in lung adenocarcinoma cancer tissue and paracancerous tissue. Brown means CD133 positive reaction, CD133 is mainly expressed in the cytoplasm, and blue means nuclear staining. All bar in the figure = 50 μm. **(B)**. GraphPad Primes 8 statistics found that CD133 expression is higher in cancer tissue than in paracancerous tissue (*p* = 0.0042). **(C)**. Western blot detects the expression of CD133 in fresh cancer tissue and paracancerous tissue of 8 lung adenocarcinoma patients. **(D)**. GraphPad Primes 8 counts the western blot test results, and it is found that compared with paracancerous tissue, CD133 expression is higher in cancer tissue (*p* = 0.00395). Bars represent the means ± SD of 8 lung adenocarcinoma patients.

As a tumor stem cell biomarker, CD133 is closely related to tumor initiation, metastasis, and recurrence. To examine the expression of CD133 in lung adenocarcinoma cancer and paracancerous tissues, we used tissue microarrays with 48 samples of lung adenocarcinoma cancer tissue paired with corresponding paracancerous tissue for IHC staining. (Figure 2A). The results showed that the localization of CD133 in cells is mainly cytoplasm, and the expression is low in lung adenocarcinoma paracancerous tissue and high in cancer tissue. The positive rate of CD133 expression in 48 cases of lung adenocarcinoma cancer tissue and paracancerous tissue was obtained by a 3D scanner, and the results showed that CD133 expression in lung adenocarcinoma cancer tissue was higher than that in paracancerous tissue (*p* = 0.042) (Figure 2B). We also collected fresh lung adenocarcinoma cancer and paracancerous tissues for Western blot analysis. The results showed that the expression of CD133 was high in lung adenocarcinoma cancer tissue and low in paracancerous tissue (*p* = 0.0395) (Figures 2C,D). These results indicated that the occurrence of lung adenocarcinoma may be associated with the overexpression of CD133.

### The Expression of XPC is Positively Correlated, while CD133 is Negatively Correlated with OS

**FIGURE 3:**
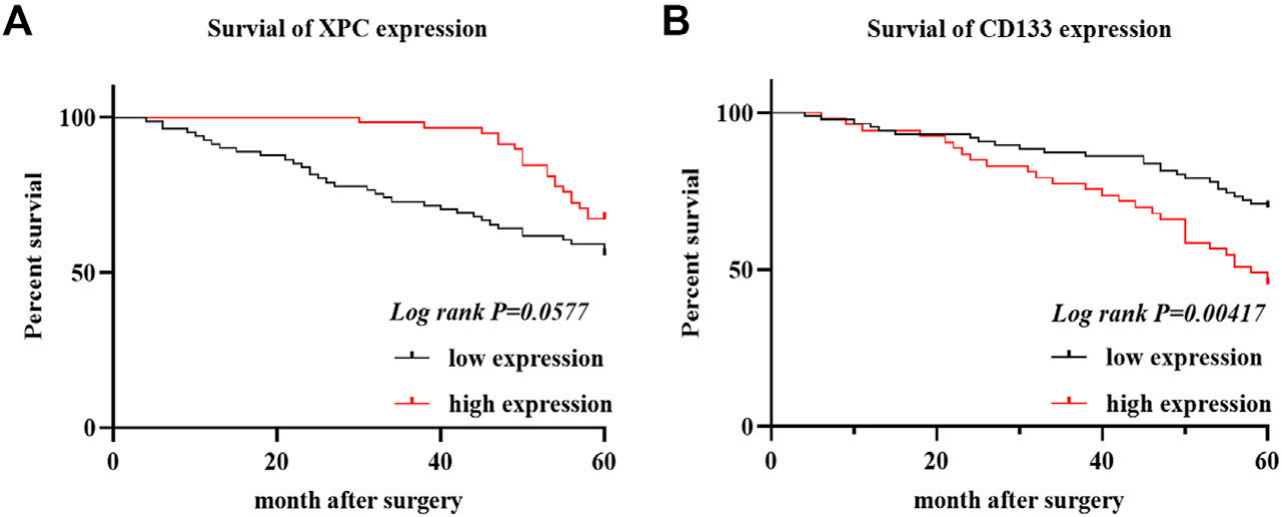
XPC and CD133 Kaplan Meier curve. **(A)**. Obtain the OS of 140 cases of lung adenocarcinoma from the HIS system, divide the expression of XPC in 140 cases of lung adenocarcinoma into two groups: XPC high expression and XPC low expression, and use Graphpad Primes 8 to analyze the survival of XPC in lung adenocarcinoma. Kaplan Meier curve found that, compared with the low XPC expression, the high XPC expression is related to the better prognosis of lung adenocarcinoma (*Log-rank p* = 0.0577). **(B)**. Divide the expression of CD133 in 140 cases of lung adenocarcinoma into two groups: high expression of CD133 and low expression of CD133. Graphpad Primes 8 was used to analyze the survival of CD133 in lung adenocarcinoma. Kaplan Meier curve found that compared with the high expression of CD133, CD133 was low. The expression is related to the better prognosis of lung adenocarcinoma (*Log-rank p* = 0.00417).

To determine the relationship between the expression of XPC and CD133 and the prognosis of lung adenocarcinoma patients, we obtained the OS of 140 enrolled lung adenocarcinoma patients through telephone follow-up and medical records. The Kaplan-Meier survival curve was used to show the relationship between XPC and CD133 positive expression rate and patient OS. The results showed that the overall survival time of lung adenocarcinoma patients with high XPC expression was longer than that of lung adenocarcinoma patients with low XPC expression (*Log-rank p* = 0.0577) (Figure 3A), and the overall survival time of lung adenocarcinoma patients with high CD133 expression was lower than that of lung adenocarcinoma patients with low CD133 expression (*Log-rank p* = 0.00417) (Figure 3B).

### The Relationship between XPC and CD133 Co-Expression and OS in Lung Adenocarcinoma Patients

**FIGURE 4:**
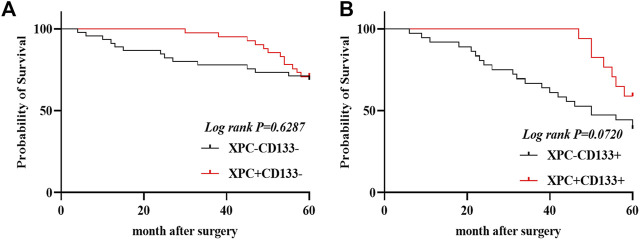
XPC and CD133 co-expression Kaplan Meier curve. **(A)**. 140 patients were divided into four groups: CD133 and XPC are both high expression: CD133+XPC+, CD133 high expression and XPC low expression: CD133+XPC−, CD133 low expression and XPC high expression: CD133−XPC+, CD133 and XPC has low expression: CD133−XPC−. Compared with the CD133+XPC− group, the CD133+XPC+ group is associated with a better prognosis of lung adenocarcinoma (*Log-rank p* = 0.6287). **(B)**. Compared with the CD133−XPC− group, the CD133-XPC + group is associated with a better prognosis of lung adenocarcinoma (*Log-rank p* = 0.0720).

The 140 lung adenocarcinoma tissues were divided into four groups based on the expression of XPC and CD133 as XPC+CD133−, XPC−CD133−, XPC+CD133+, XPC−CD133+. The results showed the OS of patients with high XPC expression showed no significant difference in low CD133 expression patients. (Figure 4A) (*Log-rank p* = 0.6287); while the OS of patients with high XPC expression in high CD133 expression patients showed a better trend, but no significant difference (Figure 4B) (*Log-rank p* = 0.0720).

### The Effect of XPC Expression on Stem Cell Biomarkers

**FIGURE 5:**
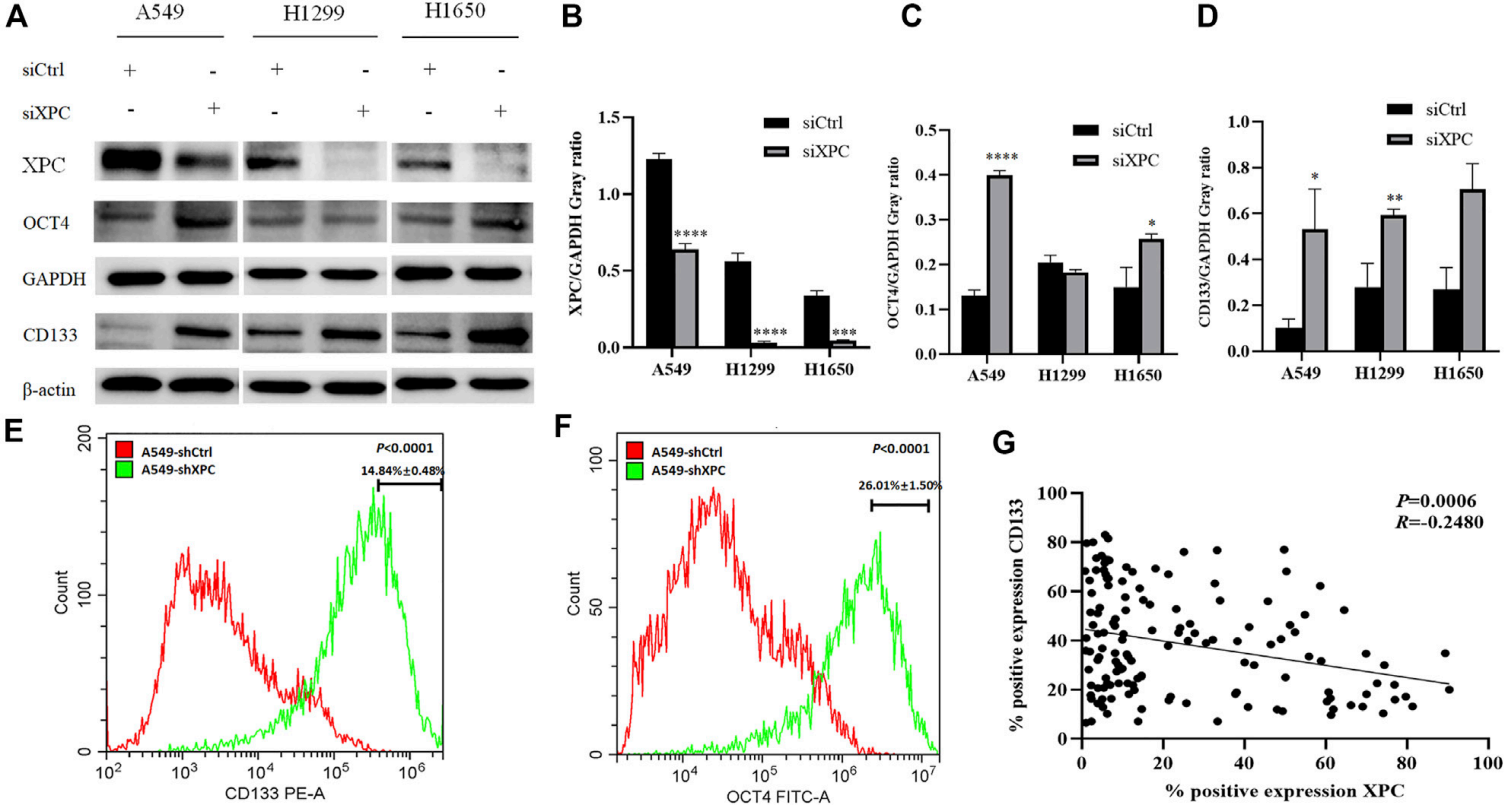
The effect of XPC expression on stem cell markers. **(A)**. The 3 cell lines A549, H1299, and H1650 were divided into two groups: siCtrl group and siXPC group. Western blot was used to detect the expression of stem cell markers in the siCtrl group and siXPC group. **(B)**. In the 3 cell lines A549, H1299, and H1650, the expression of XPC in the siXPC group was lower than that in the siCtrl group (**p < 0.01, ***p < 0.001, ****p < 0.0001). **(C)**. In the A549 and H1650 cell lines, the expression of OCT4 in the siXPC group was higher than that in the siCtrol group (*p < 0.05, ****p < 0.0001). **(D)**. In the 3 cell lines A549, H1299, and H1650, the expression of CD133 in the siXPC group was higher than that in the siCtrl group (*p < 0.05, **p < 0.01). **(E,F)**. The protein levels of CD133 and OCT4 were determined using Flow Cytometry. *p* < 0.0001(CD133), *p* < 0.0001(OCT4), A549-shXPC compared with A549-shCtrl. **(G)**. Immunohistochemical detection of XPC and CD133 expression correlation in 140 cases of lung adenocarcinoma tissues (*p* < 0.0006, *R =* −0.2580).

To test whether XPC can affect the expression of stem cell biomarkers, A549, H1299, and H1650 were knocked down the expression of XPC by siXPC, also A549 were knockeddown the expression of XPC by shXPC. Western blot showed that the expression of CD133 in the siXPC group was significantly higher than that of siCtrl in these 3 cell lines (*p < 0.05, **p < 0.01) (Figures 5A,D). Then we tested the expression of other stem cell biomarkers OCT4 in the siCtrl and siXPC groups, and found that in the A549 and H1650 cell lines, the expression of OCT4 in the siXPC group was significantly higher than that of siCtrl (*p < 0.05, **p < 0.01, ***p < 0.001, ****p < 0.0001) (Figures 5A,C). For the further exploring the regulation of XPC on CD133 and OCT4, flow cytometry was performed on A549 cells and showed that the CD133 and OCT4 positive signal intensity of shXPC group was higher than that of shCtrl (*p* < 0.0001) (Figures 5E,F). And the immunohistochemical results of cancer tissues of 140 patients with lung adenocarcinoma showed that the expression of XPC and CD133 was negatively correlated (*p* < 0.0006, *R =* −0.2580) (Figure 5G). These results demonstrated that decreased XPC expressions can increase the expression of stem cell biomarkers.

### Knockdown of XPC Promotes Lung Cancer Cell Migration

**FIGURE 6:**
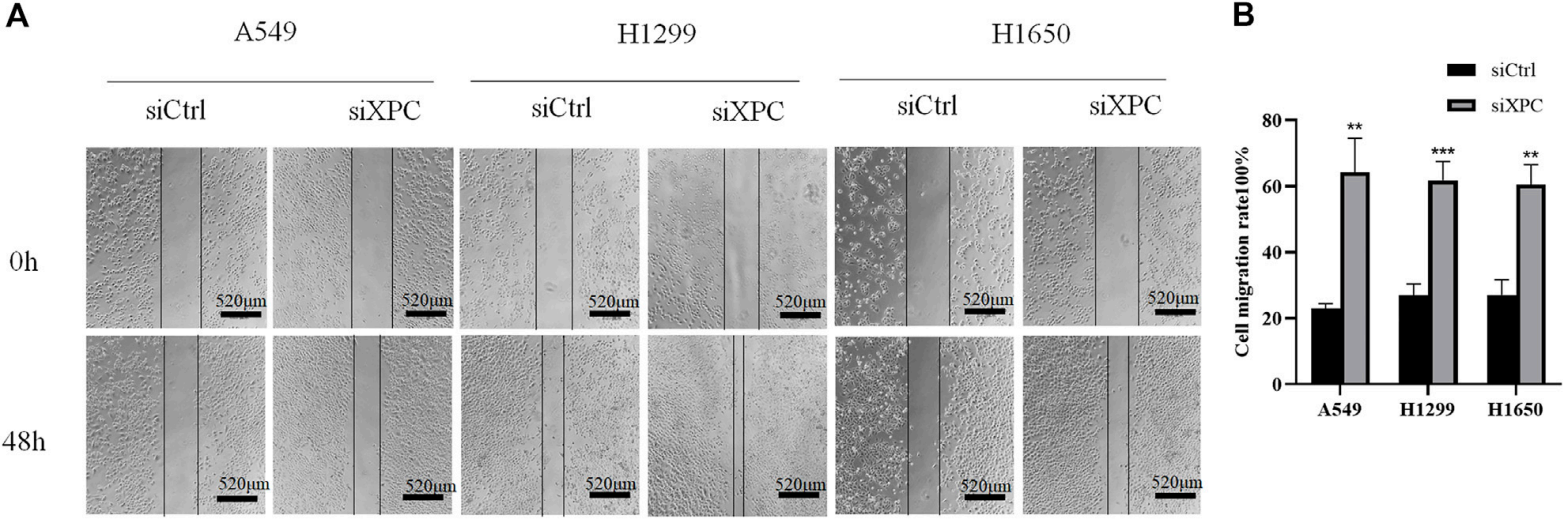
Decreased XPC expression promotes cell migration. **(A)**. Cell scratch test to detect cell migration rate. All bar in the figure = 520 μm. **(B)**. Use Image J to count the healing area of cell scratches. Cell migration rate = [(S_area at 0 h_–S_area at 48 h_)/S_area at 0 h_]×100%. The cell migration rate of the siXPC group was significantly higher than that of the siCtrl group (**p < 0.01, ***p < 0.001).

To test whether XPC can affect the migratory ability of lung cancer cells, we conducted a cell scratch experiment. The results showed that the cell migratory ability in the siXPC group was significantly greater than that in siCtrl in these 3 cell lines (**p < 0.01, ***p < 0.001) (Figure 6).

### Knockdown XPC Promotes Lung Cancer Cell Growth and Cell Stemness

**FIGURE 7:**
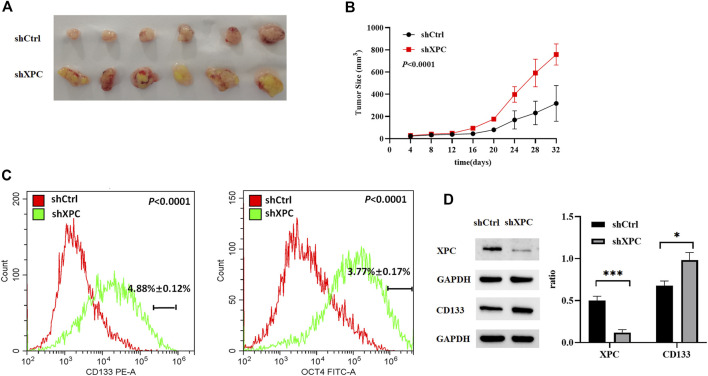
Anti-tumor effect of XPC on A549 tumor-bearing nude mice. **(A)**. Xenografts were generated by A549 cells (shCtrl), A549 cells with stable XPC knockdown (shXPC). Tumors were removed from mice after 32 days. **(B)**. Tumor sizes were measured every 4 days. **(C)**. The expression of CD133 and OCT4 were determined using Flow Cytometry. *p* < 0.0001(CD133), *p* < 0.0001(OCT4), A549-shXPC compared with A549-shCtrl. **(D)**. The expression of XPC and CD133 were determined using Western blotting. The intensity of each band was quantified using Image J and normalized to. Protein extractions come from xenotransplanted tumor tissue of mice. ***p < 0.001 (XPC), *p < 0.05(CD133), A549-shXPC compared with A549-shCtrl. (*n* = 6).

To examine the relationship between XPC expression and tumor growth and cell stemness. A549-shXPC and A549-shCtrl cells were used to generate xenografts in Athymic nude mice and tumor growth dynamics were recorded. Tumor xenografts initiated with A549-shXPC cells grew faster than those derived from A549-shCtrl cells (Figures 7A,B). Flow cytometry and Western blotting proved that the decrease of XPC expression can significantly increase the expression of lung cancer stem cell markers (Figures 7C,D). These data indicate that decreased XPC expression can increase the stemness of tumor cells and promote tumor growth.

## Discussion

XPC, as a nucleotide excision repair gene, is related to the occurrence of various cancers, such as colorectal cancer, prostate cancer, lung cancer, breast cancer, and bladder cancer (Francisco et al., 2008; Qiu et al., 2008; Peng et al., 2014; Zhu et al., 2014; Qin et al., 2020). The study by Chen et al. (2007) showed that XPC is expressed at a low level in the bladder cancer HT117 cell line. Friedberg et al. (2000) found that rats with homozygous XPC mutation after exposure to chemical carcinogens are susceptible to liver cancer. These studies showed that the XPC gene plays a very important role in DNA damage repair and tumorigenesis, and its abnormal expression was related to the susceptibility of tumors. It is speculated that XPC gene expression is also related to the occurrence of lung cancer. Viktorsson et al. (2005) and Wu et al. (2007) found that expression of XPC is low in lung cancer, and the occurrence of lung cancer is related to the low expression of XPC.

Cancer stem cells (CSC) are a small portion of cancer cells with the feature of stem cells, with the ability to self-replicate and produce heterogeneous lineages (Visvader and Lindeman, 2008; Mao et al., 2009; Tirino et al., 2013). CSCs are related to the occurrence and maintenance of tumors. In addition to the properties of self-renewal and differentiation, CSC has a high carcinogenic potential and is also known as tumor-initiating cells (Peitzsch et al., 2013; Kreso and Dick, 2014; Batlle and Clevers, 2017). Because of its high carcinogenicity, CSC is considered to be an important cause of tumor recurrence, metastasis, and drug resistance in tumor treatment (Clarke, 2005; Evers et al., 2010; Palomeras et al., 2018; Wang and Zöller, 2019; Yang et al., 2020).

The relationship between XPC and lung cancer cell stemness is unclear. In this study, we used IHC and Western blot to detect the expression of XPC and CD133 in lung adenocarcinoma cancer and paracancerous tissues. The results revealed that the expression of XPC and CD133 in lung adenocarcinoma cancer and paracancerous tissues is significantly different. The expression of XPC is significantly lower in lung adenocarcinoma cancer tissue than in paracancerous tissue. On the contrary, the expression of CD133 is significantly higher in lung adenocarcinoma cancer tissue than that in paracancerous tissue. In addition, through IHC, we found that XPC is expressed in the nucleus, while CD133 is mainly expressed in the cytoplasm. Furthermore, the expression of XPC and CD133 is correlated with the prognosis of lung adenocarcinoma. In lung adenocarcinoma patients, the expression of XPC was positively correlated with the prognosis, and the expression of CD133 was negatively correlated with the prognosis of the patients. When the expression of CD133 is the same, a higher expression of XPC is associated with a better prognosis.

Cancer stem cells not only can renew themselves but also have high carcinogenicity. The overexpression of cancer stems cell biomarkers such as CD133, OCT4, CD44, and ALDH1 is closely related to the recurrence and metastasis of cancers (Kreso and Dick, 2014; Batlle and Clevers, 2017). In our study, XPC was knocked down with siXPC in lung cancer cells A549, H1299, and H1650. The expression of stem cell biomarker CD133 was increased in these three lung cancer cell lines (A549, H1299, and H1650), while OCT4 were increased in A549 and H1650 cells, flow cytometry further proved that the expression of stem cell markers (CD133, OCT4) was significantly increased in A549-shXPC compared with A549-shCtrl. This suggesting that the decrease of XPC expression can lead to the recurrence of lung cancer by increasing the stemness of cancer cells. The cell scratch experiment also further proved that the decrease of XPC expression resulted in increasing the migration of lung cancer cells. Tumor-bearing experiments found that the decreased expression of XPC can significantly promote tumor growth, which is closely related to the regulation of the stemness of lung cancer cells by XPC.

In conclusion, our research shows that decreased expression of XPC can increase the expression of stem cell markers (CD133, OCT4), which in turn affects the proliferation and migration of lung cancer cells, then has an adverse prognosis for patients with lung adenocarcinoma.

## Data Availability

The original contributions presented in the study are included in the article further inquiries can be directed to the corresponding authors.
